# Voluntary exercise increases cholesterol efflux but not macrophage reverse cholesterol transport *in vivo *in mice

**DOI:** 10.1186/1743-7075-7-54

**Published:** 2010-07-01

**Authors:** Maxi Meissner, Niels Nijstad, Folkert Kuipers, Uwe JF Tietge

**Affiliations:** 1 Department of Pediatrics, Center for Liver, Digestive and Metabolic Diseases, University Medical Center Groningen, University of Groningen, Groningen, The Netherlands; 2 Department of Laboratory Medicine, University Medical Center Groningen, University of Groningen, Groningen, The Netherlands

## Abstract

Physical exercise beneficially impacts on the plasma lipoprotein profile as well as on the incidence of cardiovascular events and is therefore recommended in primary and secondary prevention strategies against atherosclerotic cardiovascular disease. However, the underlying mechanisms of the protective effect of exercise remain largely unknown. Therefore, the present study tested the hypothesis that voluntary exercise in mice impacts on cholesterol efflux and *in vivo *reverse cholesterol transport (RCT). After two weeks of voluntary wheel running (average 10.1 ± 1.4 km/day) plasma triglycerides were lower (p < 0.05), while otherwise lipid and lipoprotein levels did not change. Macrophage cholesterol efflux towards plasma was significantly increased in running (n = 8) compared to sedentary (n = 6) mice (14.93 ± 1.40 vs. 12.33 ± 2.60%, p < 0.05). In addition, fecal excretion of bile acids (3.86 ± 0.50 vs. 2.90 ± 0.51 nmol/d, p = 0.001) and neutral sterols (2.75 ± 0.43 vs. 1.94 ± 0.22 nmol/d, p < 0.01) was significantly higher in running mice. However, RCT from macrophages to feces remained essentially unchanged in running mice compared with sedentary controls (bile acids: 3.2 ± 1.0 vs. 2.9 ± 1.1 % of injected dose, n.s.; neutral sterols: 1.4 ± 0.7 vs. 1.1 ± 0.5 % injected dose, n.s.). Judged by the plasma lathosterol to cholesterol ratio, endogenous cholesterol synthesis was increased in exercising mice (0.15 ± 0.03 vs. 0.11 ± 0.02, p < 0.05), while the hepatic mRNA expression of key transporters for biliary cholesterol (Abcg5/g8, Sr-bI) as well as bile acid (Abcb11) and phospholipd (Abcb4) excretion did not change. These data indicate that the beneficial effects of exercise on cardiovascular health include increased cholesterol efflux, but do not extend to other components of RCT. The increased fecal cholesterol excretion observed in running mice is likely explained by higher endogenous cholesterol synthesis, however, it does not reflect increased RCT in the face of unchanged expression of key transporters for biliary sterol secretion.

## Introduction

Complications of atherosclerotic cardiovascular disease (CVD) represent a major cause of morbidity and mortality in developed societies [1]. Physical exercise is associated with a reduced risk for coronary events and is therefore recommended for primary as well as secondary prevention strategies [2,3]. As one potential beneficial effect physical exercise has been shown to improve the plasma lipoprotein profile towards a less atherogenic phenotype [4,5]. In addition, exercise in humans increases the capacity of plasma to promote cholesterol efflux from RAW-264.7 mouse macrophages *in vitro *[6]. However, the impact of exercise on *in vivo *macrophage-to-feces reverse cholesterol transport (RCT) has not been addressed. Therefore, the present study assessed the impact of voluntary exercise in mice on cholesterol efflux and *in vivo *RCT.

## Materials and methods

### Animals

Male C57BL/6J mice from Charles River (L'Arbresle, France) were kept in rooms with alternating 12-hour periods of light (from 7:00 a.m. to 7:00 p.m.) and dark (from 7:00 p.m. to 7:00 a.m.), with *ad libitum *access to water and mouse chow diet (Arie Blok, Woerden, The Netherlands). Animal experiments were performed in accordance with national laws and were approved by the responsible ethics committee of the University of Groningen.

### Voluntary cage wheel running experiments

Twelve week old mice were individually housed in cages either equipped with an 11 cm steel running wheel continuously present in the cage (running group, n = 8) or not (sedentary control group, n = 6) [7]. Distance covered and time of running were recorded daily during the two week experimental period with a digital cycling computer (K-13-TL SET-P3-NL, Xiron, The Netherlands).

### Cholesterol efflux and in vivo RCT

Thioglycollate-elicited mouse peritoneal macrophages were harvested and cultured essentially as described [8]. Macrophages were loaded for 24 h with 50 μg/ml acetylated LDL and 3 μCi/ml ^3^H-cholesterol (Perkin Elmer, Boston, MA, USA) and equilibrated for 18 h in RPMI 1640 medium containing 1% penicillin/streptomycin and 2% bovine serum albumin (Sigma, St. Louis, MO, USA). For *in vitro *cholesterol efflux experiments, cells were incubated for 24 h with 1% of respective plasma samples (performed in triplicates). Efflux was determined as the percentage of label in the supernatant related to the total amount of label within medium and cells [9]. For *in vivo *RCT two million labeled macrophages were injected intraperitoneally, blood samples were taken at 6, 24 and 48 h, feces collected for 48 h, and after 48 h livers were harvested (sacrifice by heart puncture under isoflurane anesthesia) and stored at -80°C until further analysis as previously published [9]. Plasma counts were assessed directly by liquid scintillation counting (Packard 1600CA Tri-Carb, Packard, Meriden, CT, USA). Counts within liver were determined following solubilization of the tissue (Solvable, Packard, Meriden, CT, USA) exactly as reported [10]. Fecal samples were dried, weighed and thoroughly ground. Aliquots were separated into bile acid and neutral sterol fractions as previously published [11]. Counts recovered from respective aliquots were related to the total amount of feces produced over 48 h. All obtained counts were expressed relative to the administered tracer dose.

### Plasma lipid and lipoprotein analysis

Plasma total cholesterol and triglycerides were measured enzymatically (Wako Pure Chemical Industries, Neuss, Germany). To determine plasma HDL cholesterol levels, apoB-containing lipoproteins were precipitated using 0.36% phosphotungstic acid (Sigma) and cholesterol content in the supernatant was determined as described above. Pooled plasma samples from mice of the same experimental group were subjected to fast protein liquid chromatography (FPLC) gel filtration using a Superose 6 column (GE Healthcare, Uppsala, Sweden) as described [10]. Samples were chromatographed at a flow rate of 0.5 ml/min, and fractions of 500μl each were collected. Individual fractions were assayed for cholesterol concentrations as described above. Plasma lathosterol levels relative to plasma cholesterol levels were measured by gas chromatography as described [12].

### Liver lipid analysis

Liver lipids were extracted following the general procedure of Bligh and Dyer and were determined enzymatically using commercially available reagents (Wako Pure Chemical Industries, Neuss, Germany)[13].

### Analysis of gene expression by real-time quantitative PCR

Total RNA from mouse livers was isolated using Trizol (Invitrogen), and real-time quantitative PCR was carried out on an ABI-Prism 7700 (Applied Biosystems, Foster City, CA, USA) sequence detector with the default settings [14]. PCR primers and fluorogenic probes were designed with the Primer Express Software (Applied Biosystems). mRNA expression levels were calculated relative to the average of the housekeeping gene cyclophilin and further normalized to the relative expression levels of the respective controls.

### Statistical analysis

Statistical analysis was carried out using the Statistical Package for the Social Sciences (SPSS, Inc., Chicago, IL, USA). Values are expressed as means ± SD. Student's *t *test was used to assess statistical differences between groups. Statistical significance for all comparisons was assigned at P < 0.05.

## Results

### Voluntary exercise decreases hepatic cholesterol content, while plasma cholesterol levels remain unchanged

**Figure 1:**
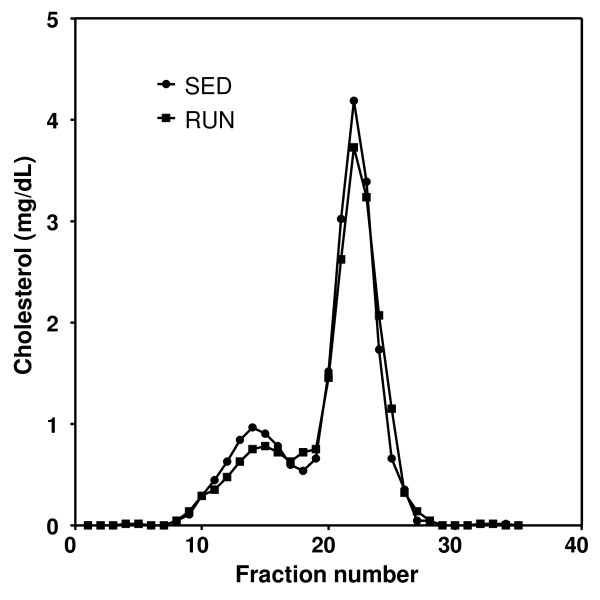
**FPLC cholesterol profiles in sedentary (SED) and exercising mice (RUN)**. Pooled plasma samples were subjected to gel filtration using a superose 6 column and cholesterol levels within each fraction were determined as described in materials and methods.

Exercising mice ran almost exclusively during the dark cycle, on average 356 ± 52 min/d thereby covering a distance of 10.2 ± 2.2 km (average speed: 1.78 ± 0.18 km/h). Plasma triglycerides were lower (p < 0.05), while phospholipids, total, non-HDL and HDL cholesterol (table 1) and apoA-I (Western blot, data not shown) remained unchanged in response to exercise. FPLC analysis revealed a small decrease in the HDL and VLDL/LDL cholesterol peaks of running mice (Figure 1). Running increased liver weight by 12% (p < 0.01, table 1), while hepatic cholesterol and triglyceride contents were decreased by 14% (p < 0.05) and 42% (p < 0.001), respectively.

**Table 1:** Plasma and liver lipids in running C57BL/6 mice compared with sedentary controls

	Sedentary (n = 6)	Running (n = 7)
PLASMA LIPIDS		
Total cholesterol (mg/dl)	85 ± 3	78 ± 7
HDL cholesterol (mg/dl)	56 ± 5	49 ± 6
Non-HDL cholesterol (mg/dl)	29 ± 4	29 ± 6
Triglycerides (mg/dl)	68 ± 20	44 ± 16*
Phospholipids (mg/dl)	182 ± 7	155 ± 23

MORPHOLOGICAL DATA		
Body weight (g)	22.2 ± 1.1	21.8 ± 1.3
Liver weight (g)	0.98 ± 0.06	1.1 ± 0.06**
Liver weight (% of body weight)	4.4 ± 0.2	5.0 ± 0.3**
Food intake (g/d)	4.1 ± 0.3	5.4 ± 0.5***

LIVER LIPIDS		
Total cholesterol (μmol/g)	7.3 ± 0.8	6.3 ± 0.7
Free cholesterol (μmol/g)	6.6 ± 0.8	5.6 ± 0.7*
Cholesterol esters (μmol/g)	0.7 ± 0.3	0.6 ± 0.1
Triglycerides (μmol/g)	24.3 ± 3.6	14.0 ± 2.3***
Phospholipids (μmol/g)	37.8 ± 5.5	33.3 ± 5.3

Values are means ± SD determined after 2 weeks of voluntary wheel running exercise. Significant differences from sedentary mice are indicated as: * p < 0.05; ** p < 0.01; *** p < 0.001.

### Cholesterol efflux from macrophage foam cells towards plasma of running mice is increased

**Figure 2:**
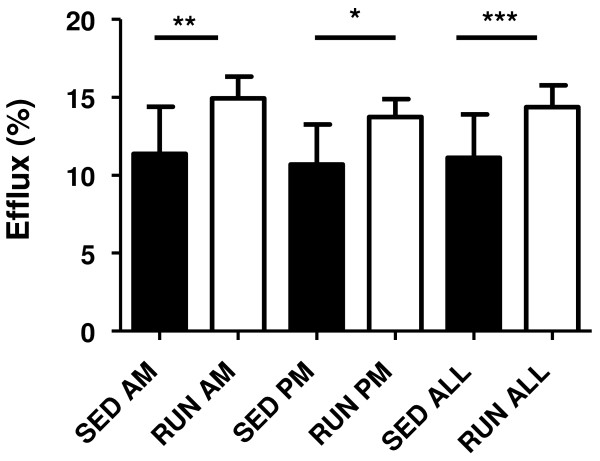
***In vitro *cholesterol efflux from macrophage foam cells towards plasma from sedentary (SED) and exercising mice (RUN)**. Efflux experiments were performed using primary mouse peritoneal macrophages and 1% plasma from the respective mice as detailed in materials and methods. Running wheels were continuously present in the cage, however, running activity occurred almost exclusively at night during the dark cycle. In order to determine, if changes in cholesterol efflux might be short-term effects occuring directly after the running period, plasma from mice bled in the morning immediately after running (AM) and bled directly before the dark cycle after rest during the day (PM) was compared separately. Data are given as means ± SD; n = 6 each for SED AM and PM; n = 9 for RUN PM; n = 8 for RUN AM; n = 12 for SED ALL and n = 17 for RUN ALL. *p < 0.05, **p < 0.01, ***p < 0.001.

Although plasma total cholesterol levels did not change and HDL cholesterol levels tended to be lower in running mice, *in vitro *cholesterol efflux towards plasma of the running mice was significantly increased (p < 0.001, Figure 2). This effect was consistent independent of the blood sampling time from these mice, either directly after (a.m.) or before (p.m.) the running period.

### Macrophage-to-feces RCT is unchanged in exercising mice

**Figure 3:**
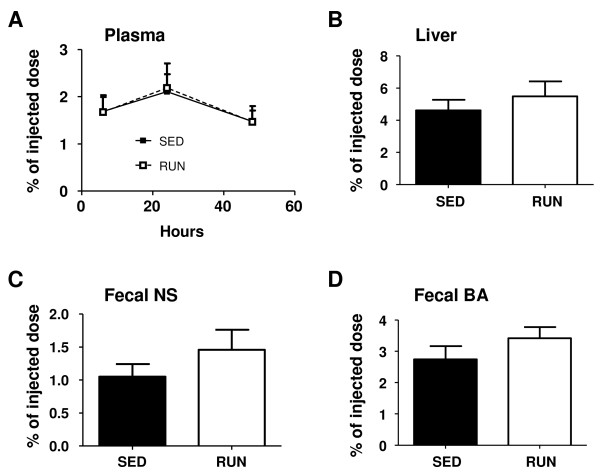
***In vivo *reverse cholesterol transport in sedentary (SED) and exercising (RUN) mice**. At the end of the 2-week experimental period exercising mice (n = 8) and sedentary controls (n = 6) were injected intraperitoneally with ^3^H-cholesterol-labeled and acLDL-loaded primary mouse peritoneal macrophages and tracer appearance was followed for 48 h as detailed in materials and methods. (A) Time course of tracer appearance in plasma, (B) tracer recovery within liver at the 48 h time point, (C) tracer level within fecal bile acids (BA) after 48 h, (D) tracer recovery within fecal neutral sterols (NS) after 48 h. Data are expressed as percentage of the injected tracer dose and are given as means ± SD.

**Figure 4:**
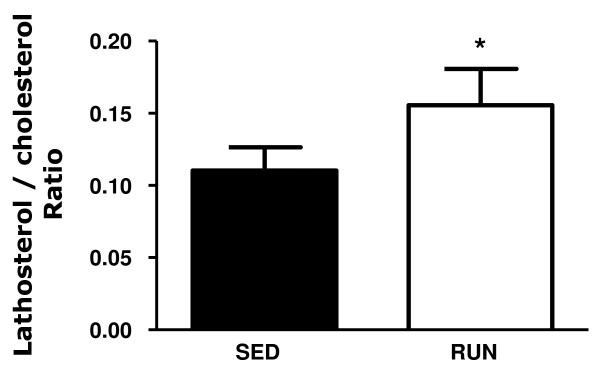
**Effect of two week voluntary wheel running on the plasma lathosterol/cholesterol ratio as marker of endogenous cholesterol synthesis in exercising mice (RUN, n = 8) and sedentary controls (SED, n = 6)**. Plasma cholesterol and lathosterol levels were determined by gas chromatography as detailed in materials and methods. Data are given as means ± SD, *p < 0.05.

Next, *in vivo *RCT experiments were performed. ^3^H-cholesterol originating from macrophages was unchanged in plasma comparing running with sedentary mice as were counts within liver (Figure 3). Daily feces production (871 ± 97 vs. 616 ± 54 mg/d, p < 0.001) and mass fecal excretion of bile acids (3.86 ± 0.50 vs. 2.90 ± 0.51 nmol/d, p = 0.001) and neutral sterols (2.70 ± 0.25 vs. 1.90 ± 0.39 nmol/d, p < 0.01) were increased in the running group. However, fecal excretion of ^3^H-cholesterol tracer originating from macrophages, reflecting completed RCT, remained unchanged both within neutral sterols and bile acids (Figure 3). In support of these physiological data, also the mRNA expression of several transporters critical for the biliary secretion of cholesterol (Abcg5/g8, Sr-bI), bile acids (Abcb11) and phospholipids (Abcb4) remained unchanged (table 2). The plasma lathosterol/cholesterol ratio as a measure of endogenous cholesterol synthesis was significantly higher in the running mice (Figure 4) indicating that the increased amount of fecal sterols secreted in this group is rather originating from increased cholesterol synthesis than reflecting increased RCT.

**Table 2:** Hepatic gene expression levels in running C57BL/6 mice compared with sedentary controls

	Sedentary (n = 6)	Running (n = 7)
Abcg5	1.00 ± 0.3	1.2 ± 0.3
Abcg8	1.00 ± 0.2	1.3 ± 0.3
Sr-bI	1.00 ± 0.1	1.0 ± 0.2
Abcb11	1.00 ± 0.1	1.1 ± 0.2
Abcb4	1.00 ± 0.2	1.1 ± 0.2

mRNA expression levels were determined by real-time quantitative PCR in livers of C57BL/6 mice after 2 weeks of voluntary wheel running exercise compared with sedentary controls. Results are normalized to the expression of the housekeeping gene cyclophilin and are expressed relative to the respective controls. Data are given as means ± SD.

## Discussion

The results of this study demonstrate that voluntary exercise in mice increases the efflux capacity of plasma despite a tendency towards decreased plasma HDL cholesterol levels, but does not alter macrophage-to-feces RCT. To the best of our knowledge this is the first study investigating a potential impact of exercise on *in vivo *RCT. While the beneficial effects of exercise on cardiovascular health have long been noted and exercise has been implemented in the recommendations for primary as well as secondary prevention strategies [3], the precise underlying mechanism for exercise decreasing CVD risk has not been fully elucidated thus far. It has been noted that aerobic capacity training decreases markers of inflammation and oxidative stress as well as blood pressure levels over time [3,15-20], while acute endurance exercise such as marathon running is associated with increased oxidative stress and a pro-inflammatory response [21,22]. As a further beneficial effect of exercise in humans increased plasma HDL cholesterol levels and an increased capacity of plasma from exercising individuals to stimulate cholesterol efflux from macrophage foam cells *in vitro *has been noted [6]. These results have been related to an increased plasma level of preβ-HDL particles in trained individuals [23,24]. Our study confirmed the efflux data in a mouse model of voluntary endurance exercise, while in contrast to the human situation plasma HDL cholesterol levels rather had a tendency to be lower in exercising mice. The increase in cholesterol efflux *in vitro *was not reflected by altered plasma counts in the *in vivo *RCT experiment, likely since these represent the net balance between efflux and removal from the plasma compartment. Interestingly, feces production was increased in exercising mice, which also mirrors the human situation [25]. In addition, fecal mass secretion of bile acids and neutral sterols was significantly elevated in response to exercise, which in our interpretation reflects increased endogenous hepatic cholesterol synthesis. In humans one study reported a non-significant 63% increase in endogenous cholesterol synthesis using the deuterium incorporation method [26], while another study detected no difference in plasma lathosterol levels in response to exercise training [27]. Since RCT in humans differs in several aspects from mice (e.g. by the expression of CETP), studies on the impact of exercise on RCT in humans will be interesting to perform, once an integrated experimental system for these types of studies becomes available.

## Competing interests

The authors declare that they have no competing interests.

## Authors' contributions

MM and NN were involved in the acquisition and analysis of the data, participated in the design of the study and drafted the manuscript. FK contributed to interpretation of the data and critical revision of the manuscript. UJFT conceived of the study, participated in its design and coordination, and critically revised the manuscript. All authors read and approved the final manuscript.
